# Pathways of Oxygen-Dependent Oxidation of the Plastoquinone Pool in the Dark After Illumination

**DOI:** 10.3390/plants13243479

**Published:** 2024-12-12

**Authors:** Ilya Naydov, Marina Kozuleva, Boris Ivanov, Maria Borisova-Mubarakshina, Daria Vilyanen

**Affiliations:** Institute of Basic Biological Problems of the Russian Academy of Sciences, Federal Research Center “Pushchino Scientific Center for Biological Research of the Russian Academy of Sciences”, 142290 Pushchino, Russia; eliotfur@gmail.com (I.N.); ivboni@rambler.ru (B.I.); mubarakshinamm@gmail.com (M.B.-M.)

**Keywords:** photosynthesis, plastoquinone, hydrogen peroxide, PTOX, OJIP

## Abstract

The redox state of the plastoquinone (PQ) pool in thylakoids plays an important role in the regulation of chloroplast metabolism. In the light, the PQ pool is mostly reduced, followed by oxidation after light cessation. It has been believed for a long time that dark oxidation depends on oxygen, although the precise mechanisms of the process are still unknown and debated. In this work, we analyzed PQ pool oxidation kinetics in isolated pea (*Pisum sativum*) thylakoids by tracking the changes in the area above the OJIP fluorescence curve (A_fl_) over time intervals from 0.1 s to 10 min in the dark following illumination. A_fl_ served as an indirect measure of the redox state of the PQ pool that enabled quantification of the rate of PQ pool oxidation. The results showed a two-phase increase in A_fl_. The “fast” phase appeared to be linked to electron flow from the PQ pool to downstream acceptors of the photosynthetic electron transport chain. The “slow” phase involved oxidation of PQH_2_ through oxygen-dependent mechanisms. Adding octyl gallate, an inhibitor of plastid terminal oxidase (PTOX), to isolated thylakoid suspensions decreased the rate of the “slow” phase of PQ pool oxidation in the dark after illumination. The addition of either H_2_O_2_ or catalase, an enzyme that decomposes H_2_O_2_, revealed that H_2_O_2_ accelerates oxidation of the PQ pool. This indicates that under conditions that favor H_2_O_2_ accumulation, H_2_O_2_ can contribute substantially to PQ pool oxidation in the dark after illumination. The contribution of PTOX and H_2_O_2_ to the modulation of the PQ pool redox state in plants in the dark after illumination is discussed.

## 1. Introduction

The plastoquinone (PQ) pool is a central component of the photosynthetic electron transport chain (PETC) in chloroplasts of higher plants. Reduced PQ molecules, plastohydroquinone (PQH_2_), transfer electrons between photosystem II (PS II) and the cytochrome b_6_f complex during linear electron transport and contribute to the generation of a proton gradient across the thylakoid membrane. The PQ pool components are also involved in cyclic electron transport around photosystem I (PS I), the Mehler reaction [1], and chlororespiration [2]. PQ pool molecules perform an antioxidant function, neutralizing the reactive oxygen species (ROS) formed during photosynthesis [3,4,5] and terminating lipid peroxidation [6]. Additionally, numerous studies [7,8,9,10,11] indicate that the redox state of the PQ pool is involved in activating signaling pathways that help plants acclimate to environmental conditions.

In nature, plants may experience abrupt changes in light conditions, such as shading, and investigating the pathways of PQ pool oxidation in darkness following illumination is crucial for understanding how plants acclimate to changing light conditions. However, the specific pathways of PQ pool oxidation in darkness after illumination are not fully understood. Under illumination, the oxidation of the PQ pool is primarily carried out by the cytochrome b_6_f complex, which supplies PS I with electrons via plastocyanin. Upon the cessation of light, the oxidized PS I centers contribute to the oxidation of the PQ pool. However, the number of PQ molecules in the pool largely exceeds the number of major photosynthetic complexes [12]. Therefore, not all PQ molecules are oxidized by PS I centers in darkness.

It has been known for a long time that oxygen is required for PQ pool oxidation in darkness after illumination [13]. The oxidation of PQH_2_ by O_2_ is believed to proceed via an autocatalytic mechanism [14,15]. Plastosemiquinone, formed in the disproportionation reaction of PQ and PQH_2_, interacts with O_2_, leading to the formation of superoxide anion radicals, which can subsequently oxidize PQH_2_ [3].

PTOX (plastid terminal oxidase) is another potential participant in the oxidation of the PQ pool in darkness following illumination. PTOX catalyzes the transfer of electrons from PQH_2_ to O_2_ with H_2_O formation [16]. It has been shown that PTOX is necessary for chloroplast maturation during leaf development [17,18,19] and for carotenoid synthesis during the formation of the photosynthetic apparatus [20,21,22]. PTOX, together with the NDH complex, participates in chlororespiration [22,23,24]. However, its role in the dark oxidation of the PQ pool in mature chloroplasts of higher plants has not been proven. Arabidopsis IMMUTANS lines, which lack PTOX, have shown slower PQ pool oxidation in darkness following illumination [2,25,26]. However, in mature chloroplasts of higher plants, the PTOX content is low: the PTOX:PS II ratio is about 1:100 [27], and the rate constant for PQH_2_ oxidation by O_2_ catalyzed by PTOX in the membrane is only 10^1^–10^2^ m^−1^s^−1^ [14,28]. Furthermore, in IMMUTANS plants lacking PTOX during plastid development, the formation of the photosynthetic apparatus is disrupted, potentially triggering compensatory mechanisms, complicating the interpretation of observed effects. Finally, it is possible that the mitochondrial alternative oxidase, a related enzyme, might be transported to chloroplasts in the absence of PTOX and partially perform its function [29].

The PQ pool can also be oxidized by the ROS generated during the interaction of the photosynthetic apparatus with oxygen molecules in the light. The PQ pool can react with superoxide anion radicals [3,30,31] formed in the PQ pool or in PS I and with singlet oxygen [4,5,32,33]. It has also been suggested that the PQ pool may interact with H_2_O_2_ [3,14]. One study [34] demonstrated the interaction of semiquinones with H_2_O_2_ in a DMSO/phosphate buffer. However, evidence that the PQ pool is oxidized by H_2_O_2_ molecules produced in the PETC in the light has not yet been obtained.

PQ pool oxidation in darkness has been previously studied in isolated thylakoids and intact leaves using spectroscopy and fluorescence techniques [2,35,36,37]. The fast chlorophyll *a* fluorescence induction transients (OJIP curves) allow for the assessment of electron transfer events across the entire photosynthetic electron transport chain. In intact thylakoids where efficient electron acceptors from PS I are absent, applying a saturating flash causes electrons to accumulate, leading to a reduction in the PQ pool and downstream acceptors. Because chlorophyll fluorescence yield inversely reflects the availability of oxidized electron acceptors from PS II, this accumulation results in increased fluorescence. The area above the OJIP curve integrates these fluorescence changes over time, providing a cumulative measure of electron carrier availability. Therefore, analyzing this area offers a reliable indirect measure of the amount of available oxidized electron carriers in the PETC, primarily the oxidized PQ [37].

In this study, we investigated oxygen-dependent pathways of PQ pool oxidation in darkness by measuring changes in the area above the OJIP curves at various time intervals in darkness after illumination. Isolated pea (*Pisum sativum*) thylakoids were used as an experimental model. We showed that PQ pool oxidation in darkness is facilitated by both H_2_O_2_ accumulated in the light and PTOX. The conditions under which these pathways dominate are also discussed.

## 2. Materials and Methods

### 2.1. Plant Material

Pea plants (*Pisum sativum*), aged 1.5–2 weeks, grown in a greenhouse at 21–23 °C, were used for the thylakoid isolation and OJIP measurements. Commercially available fresh spinach leaves were used for the chloroplast isolation. Arabidopsis plants (*Arabidopsis thaliana*) were grown in a growing chamber (100 µmol photons m^−2^s^−1^ light intensity and an 8 h day/16 h night photoperiod) and used for the thylakoid isolation.

### 2.2. Thylakoids and Chloroplasts Isolation

Thylakoids from pea leaves were isolated according to [15], using leaves from the first and second layers. The isolated thylakoids were resuspended in a medium containing 0.4 M sucrose, 20 mM NaCl, 5 mM MgCl_2_, and 50 mM HEPES-KOH (pH 7.6). They were stored on ice in the dark and used the same day. Intact thylakoids from *Arabidopsis thaliana* of the wild type were isolated according to [38]. Thylakoids were resuspended in a solution containing 0.4 M sorbitol, 10 mM NaCl, 5 mM MgCl_2_, 2.5 mM Na_2_-EDTA, and 20 mM HEPES-KOH (pH 7.6), kept in the dark on ice, and used the same day.

Intact chloroplasts were isolated from fresh spinach leaves according to [39] and resuspended in a medium containing 0.33 M sorbitol, 60 mM KCl, 2 mM EDTA, 1 mM MgCl_2_, 0.5 mM KH_2_PO_4_, and 25 mM HEPES-KOH (pH 7.6). The intactness of the chloroplasts was assessed by comparing oxygen evolution rates in the presence of 10 mM NH_4_Cl (uncoupler) and 1 mM K_3_[Fe(CN)_6_] (electron acceptor from PETC) before and after disruption of the chloroplasts according to [40], and in all experiments, intactness was at least 90%.

Chlorophyll was extracted in 96% ethanol, and the chlorophyll (a + b) concentration was determined according to [41].

The reaction medium for experiments with isolated thylakoids included 0.1 M sucrose, 20 mM NaCl, 5 mM MgCl_2_, 50 mM HEPES-KOH (pH 7.6), and 1 µM Gramicidin D (GrD) as the uncoupler. The reaction medium for experiments with isolated chloroplasts included 0.33 M sorbitol, 60 mM KCl, 2 mM EDTA, 1 mM MgCl_2_, 0.5 mM KH_2_PO_4_, 25 mM HEPES-KOH (pH 7.6), and 10 mM NH_4_Cl.

### 2.3. Measurement of OJIP Curves

OJIP curves in leaves, isolated chloroplasts, and thylakoids were measured using the HandyPea device (Hansatech, Pentney, UK) in the dark at various time intervals following illumination. Conditions of pre-illumination were varied. A single saturating red-light flash (λ = 660 nm) at an intensity of 3000 µmol quanta m^−2^ s^−1^ for 1.5 s was used to fully reduce the PQ pool in both dark-adapted and pre-illuminated samples immediately before turning off the light to standardize the redox state of the PQ pool prior to tracking its oxidation in the dark. A new sample or leaf was used for each dark interval from 0.1 to 600 s. In other words, they were illuminated with the flash twice: once to reduce the PQ pool and a second time after a set dark period to measure the OJIP curve and estimate the area above the OJIP fluorescence curve (A_fl_). At least three leaves or suspensions were used for each time point of each experiment.

A_fl_ was used as an indirect measure of the PQ pool redox state. The higher the A_fl_, the more oxidized the PQ pool, and conversely, the lower the A_fl_, the more reduced the PQ pool. The dependence of A_fl_ on dark time was plotted and fitted using the Origin software 2021. The following function was used to fit the biphasic dependence of A_fl_ on dark time:

$$A_{fl}\left(t\right)=A_{total}-A_{1}\times e^{-k_{1}t}-A_{2}\times e^{-k_{2}t}$$

where t is the time in darkness; A_total_ is the total amplitude of A_fl_ changes; A_1_ and A_2_ are the amplitudes of the first and second phases of A_fl_ changes; and k_1_ and k_2_ are the rate constants of these phases. The half-times of the first (τ_1_) and second (τ_2_) phases were calculated:

$$\tau_{n}=\frac{1}{k_{n}}\times \ln2$$

### 2.4. Electron Transport Rate Estimation

The electron transport rate in thylakoids was measured as light-induced net oxygen uptake in the presence of 1 µM GrD and 50 µM methyl viologen using a Clark-type oxygen electrode (Hansatech, Pentney, UK).

### 2.5. Estimation of Inhibition of PS II Activity by H_2_O_2_

To evaluate the effect of H_2_O_2_ concentration on PS II oxygen-evolving activity, thylakoids were incubated with H_2_O_2_ at concentrations of 0.05, 0.1, 0.5, and 5 mM with stirring for 60 s in darkness following a saturating flash. The thylakoids were then separated from the H_2_O_2_ by centrifuging the suspension at 1600× *g* for 3 min. Next, the thylakoids were resuspended in a standard storage medium (see Section 2.2). PS II activity was assessed by measuring light-induced oxygen evolution in the presence of 1 µM GrD, 50 µM 2,6-dichloro-1,4-benzoquinone (DCBQ), and 5 mM K_3_[Fe(CN)_6_] as an electron acceptor pair.

### 2.6. Estimation of Membrane-Bounded PTOX Level in Thylakoids with Western Blot with Antibodies Against PTOX

*Arabidopsis* thylakoid suspensions were illuminated with a saturating flash of 1.5 s duration (SP) or with high-intensity continuous light (HL, approximately 650 μmol quanta m^−2^s^−1^) for 30 s. After illumination, the thylakoid suspensions were centrifuged at 10,000× *g* for 3 min to remove dissociated membrane components. The supernatant was discarded, and the pellet was resuspended in the storage buffer. As a control, thylakoid suspensions were subjected to the same manipulations but were kept in the dark. Untreated thylakoids were also used as a reference.

The PTOX level in *Arabidopsis* thylakoids was determined using immunoblotting. Samples were incubated with 2% (*w*/*v*) SDS and 100 mM DTT at 99 °C for 3 min and separated on 15% SDS-PAGE in Mini-PROTEAN Tetra Cell (Bio-Rad, Hercules, CA, USA). Protein molecular weight markers (Blue Plus Protein, TransGen Biotech, Beijing, China) were used to estimate protein size. After electrophoresis, the proteins were transferred to polyvinylidene fluoride (PVDF) membrane (Bio-Rad, Hercules, CA, USA) using a wet blotting system (Mini Trans-Blot Cell, Bio-Rad, Hercules, CA, USA). The membrane was blocked with 2% (*w*/*v*) non-fat dry milk in buffer containing 20 mM Tris-HCl (pH 7.6) and 125 mM NaCl, following incubation with primary polyclonal rabbit antibodies specific to PTOX (AS23 4895, Agrisera, Vännäs, Sweden) overnight at 4 °C. Goat anti-rabbit IgG conjugated with alkaline phosphatase (Bio-Rad, Hercules, CA, USA) was used as the secondary antibody. Protein bands were visualized using an alkaline phosphatase conjugate substrate kit (Bio-Rad, Hercules, CA, USA), following the manufacturer’s protocol. The 37 kDa band was taken as the PTOX band, according to Agrisera information.

### 2.7. Statistical Analysis

The significance of differences between the half-times (τ_1_ and τ_2_) of A_fl_ kinetics under different conditions was assessed using a *t*-test with a significance threshold of *p* < 0.05. Statistical analysis was performed using the Origin software package. The significant difference between H_2_O_2_ concentration variants was calculated by the Holm–Bonferroni test (*p* < 0.05).

### 2.8. Chemicals

To create an efficient electron flow from PS I, 500 µM NADP^+^ and 10 µM recombinant ferredoxin (petF from *Chlamydomonas reinhardtii*) obtained via the heterologous expression in *Escherichia coli* BL21 and purified according to [42] were used. Anaerobic conditions in thylakoid or chloroplast suspensions were generated using 50 units/mL glucose oxidase, 20 mM glucose, and 250 units/mL catalase. BSA was used in amounts corresponding to the catalase concentration (0.07 mg/mL) as a control to account for nonspecific protein effects. Octyl gallate (Sigma-Aldrich, St. Louis, MO, USA) was used to assess the impact of PTOX. 2,4-dinitrophenyl ether of 2-iodo-4-nitrothymol (DNP-INT) (Cayman Chemical, Ann Arbor, MI, USA) was used to inhibit PQH_2_ molecules’ oxidation at the Qo site of the cytochrome b_6_f complex.

## 3. Results

### 3.1. Isolated Thylakoids as an Experimental Model for Studying Oxygen-Dependent Pathways of PQ Pool Oxidation in Darkness After Illumination

**Figure 1:**
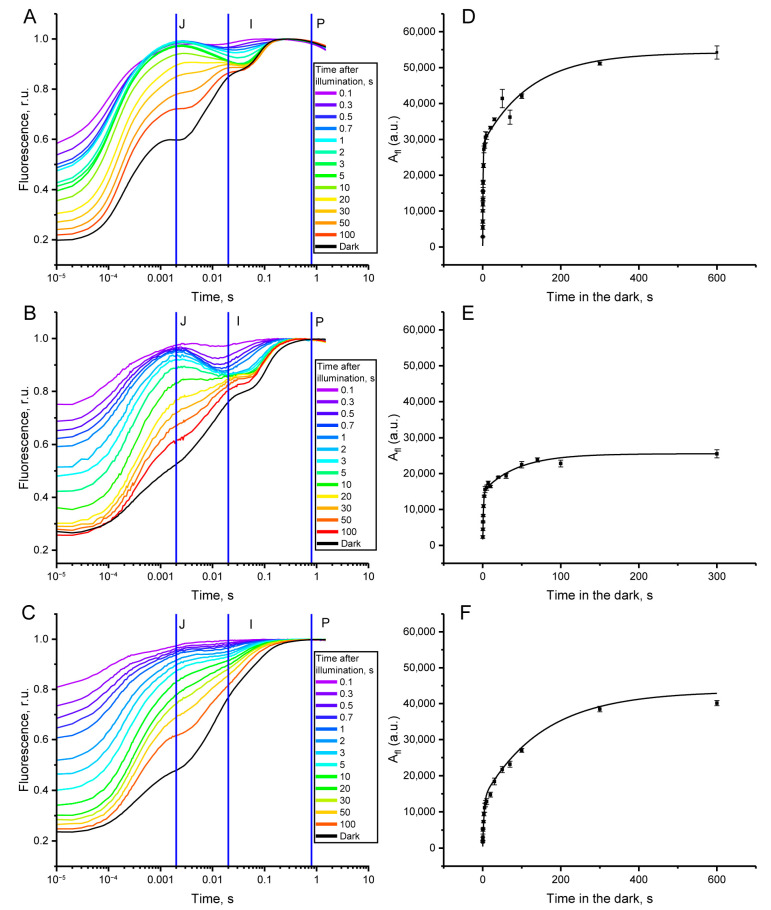
OJIP kinetics measured at various time intervals in darkness after illumination with a saturating flash in pea leaves (**A**), isolated spinach chloroplasts (**B**), and isolated pea thylakoids with oxygen as the only electron acceptor (**C**). Time dependencies of A_fl_ in darkness after illumination with a saturating flash in pea leaves (**D**), isolated spinach chloroplasts (**E**), and isolated pea thylakoids with oxygen as the only electron acceptor (**F**).

In the first phase, isolated pea thylakoids were validated as a model system for investigating oxygen-dependent pathways of PQ pool oxidation in darkness after illumination using the OJIP curve analysis. OJIP curves measured at various time intervals in darkness after exposure to a single saturating light flash (1.5 s of 3000 µmol quanta m^−2^ s^−1^) in pea leaves (Figure 1A), isolated spinach chloroplasts (Figure 1B), and isolated pea thylakoids with oxygen as the only electron acceptor (Figure 1C), were analyzed. All sets of OJIP curves, measured after the light flash, showed a tendency to return over time in darkness to the shape measured in dark-adapted samples (Figure 1A–C, black line), reflecting the gradual oxidation of PETC components in darkness, particularly of the PQ pool. However, the specific patterns of OJIP shape changes were revealed when comparing leaves, isolated chloroplasts, and thylakoids (Figure 1A–C).

The most pronounced differences between leaves and isolated chloroplasts versus thylakoids were observed in the J-I and I-P phases, especially within the first second of darkness after illumination. According to classical interpretations of the JIP-test, the J-I phase represents the reduction in the PQ pool, while the I-P phase reflects electron flow from the PQ pool to downstream acceptors of PETC [43]. This suggests that the marked “dip” in fluorescence yield in these regions of the OJIP curves, observed in leaves and chloroplasts, is due to the presence of an efficient electron acceptor that, in light, ensures a high level of oxidized P700 and plastocyanin, leading to efficient PQ pool oxidation during the first second of darkness. In contrast, thylakoids with oxygen as the only electron acceptor showed almost no fluorescence “dip” in the initial moments, though significant changes occurred in the J-I region after 1 s of darkness. Unexpectedly, the addition of ferredoxin (Fd) and NADP^+^ to the thylakoid suspension, intended to simulate efficient electron acceptors, as in chloroplasts, did not lead to significant differences in the OJIP curve patterns compared to thylakoids without acceptors after the saturating light flash (Supplementary Figure S1A). However, after illuminating the thylakoid suspension with high light for 30 s in the presence of Fd and NADP^+^, a pronounced fluorescence “dip” was observed in the first seconds of darkness (Supplementary Figure S1B), characteristic of leaves and isolated chloroplasts, indicating the presence of an effective electron acceptor in the thylakoid PETC.

**Figure 2:**
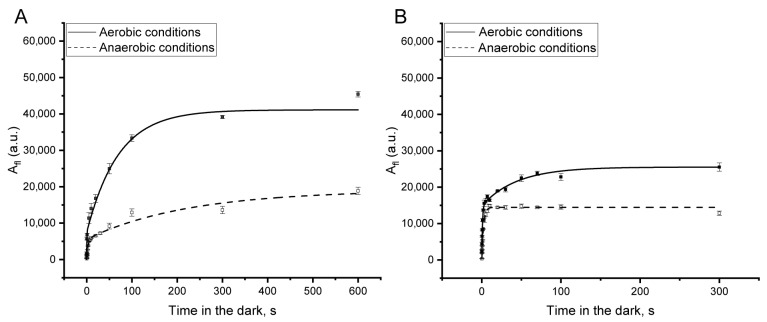
Dependencies of A_fl_ on time intervals in darkness after illumination with a single saturating flash in isolated pea thylakoids (**A**) and spinach chloroplasts (**B**) under aerobic (solid line) and anaerobic (dashed line) conditions.

The area above the fluorescence curve (A_fl_) was calculated from the measured OJIP curves. A_fl_ depends on changes in the curve shape, particularly in the J-I and I-P phases. The dependence of A_fl_ on the time in darkness (hereafter referred to as A_fl_ kinetics) for each variant is shown in Figure 1D–F. For all samples, A_fl_ kinetics exhibited a biphasic pattern with distinct “fast” and “slow” phases. However, the relative contributions of these phases varied among the samples. Leaves and isolated chloroplasts displayed significant changes in OJIP curve shape in the initial moments of darkness, reflected as a larger (approximately 50%) contribution of the “fast” phase to the total amplitude of the A_fl_ changes (Figure 1D,E). In isolated thylakoids, however, the contribution of the “fast” phase was lower (approximately 25%), making the “slow” phase the main contributor to the A_fl_ changes (Figure 1F). In separate experiments performed under anaerobic conditions (provided by glucose, glucose oxidase, and catalase), significant suppression of the “slow” phase of A_fl_ kinetics was observed in thylakoids (Figure 2A), while this phase was fully suppressed in chloroplasts (Figure 2B). The “fast” phase was mostly unaffected by anaerobic conditions. This indicates the involvement of oxygen primarily in the “slow” phase of A_fl_ kinetics in thylakoids and chloroplasts in darkness.

Thus, isolated thylakoids can serve as a valid experimental model for studying oxygen-dependent mechanisms of PQ pool oxidation through A_fl_ kinetics measurements. The use of isolated thylakoids eliminates the influence of processes such as state transitions (in the absence of ATP addition) and cyclic electron transport around PSI (in the absence of Fd and NADP^+^ addition). Moreover, the addition of the uncoupler, gramicidin D, to the medium prevents the development of non-photochemical quenching, which could otherwise affect Fm.

### 3.2. Effect of Continuous and Single Flash Pre-Illumination on PQ Pool Oxidation in Darkness

The effects of two types of pre-illumination on A_fl_ kinetics in darkness—continuous pre-illumination and a single saturating flash—were compared. For this, thylakoid suspensions were pre-illuminated with a single saturating flash for 1.5 s (3000 µmol quanta m^−2^ s^−1^) or continuous high light (650 µmol quanta m^−2^ s^−1^) for 30 s, followed by OJIP kinetics measurements in the dark as described previously. The half-times of the “fast” (τ_1_) and “slow” (τ_2_) phases were calculated to analyze A_fl_ kinetics (see Materials and Methods, Section 2.3). As shown in Table 1, after continuous pre-illumination, τ_2_ decreased compared to flash pre-illumination, while τ_1_ remained unchanged. After pre-illumination with high light (1000 µmol quanta m^−2^ s^−1^), a smaller τ_2_ value was observed compared to low light (10 µmol quanta m^−2^ s^−1^), while τ_1_ remained constant (Table 1).

Thus, continuous pre-illumination alters the state of the PETC, accelerating the increase in A_fl_. Moreover, this effect is more pronounced at higher light intensity.

### 3.3. Effect of Octyl Gallate, a PTOX Inhibitor, on PQ Pool Oxidation in Darkness After Illumination

PTOX catalyzes electron transfer from PQH_2_ to O_2_, producing H_2_O, and is proposed to participate in the dark oxidation of the PQ pool in thylakoids [2]. PTOX involvement in A_fl_ kinetics was investigated using octyl gallate (OG), a widely used PTOX inhibitor.

**Figure 3:**
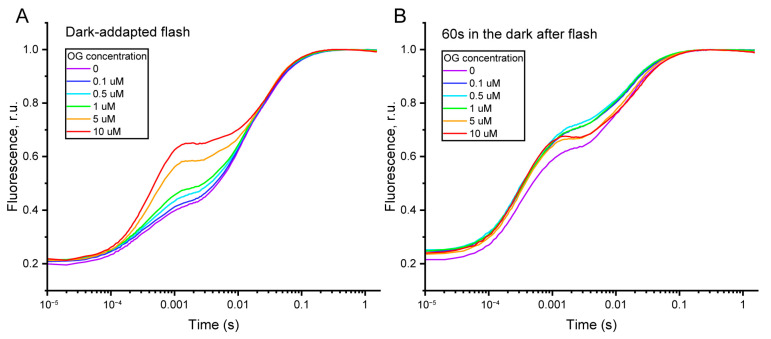
Concentration effect of OG on OJIP curve shape in dark-adapted pea thylakoids (**A**) and after 60 s of darkness following illumination of pea thylakoids with a saturating flash (**B**).

In the first step, we selected the optimal OG concentration. In the range of 1–100 µM OG, no effect on the net electron transport rate was observed, measured in thylakoid suspensions in the presence of gramicidin D and methyl viologen using a Clark-type oxygen electrode (Supplementary Figure S2). However, at 0.5 µM OG, changes in the O-J and J-I phases of the OJIP curve, measured in dark-adapted thylakoids, were observed (Figure 3A). A further increase in OG concentration resulted in a significant elevation of the F_J_ level. Simultaneously, we evaluated which OG concentrations slowed the increase in A_fl_ in isolated thylakoids after 60 s of darkness following illumination (Figure 3B). The greatest differences were observed at 0.1, 0.5, and 1 µM OG, while the OJIP curve shape at 5 and 10 µM OG differed little from the control, suggesting that these concentrations of OG either do not affect PQ pool oxidation or exhibit nonspecific activity. As a result, we selected 1 µM OG, which has been shown to suppress recombinant PTOX activity by 80% [44].

At 1 µM OG, τ_2_ increased nearly threefold compared to the control after illumination with a single saturating flash, while τ_1_ remained unchanged (Table 2). This confirms the involvement of PTOX in PQ pool oxidation in thylakoids in darkness after illumination. This result also demonstrates that PQ pool oxidation makes a significant contribution to the observed A_fl_ kinetics in thylakoids. However, after continuous pre-illumination with high light (650 µmol quanta m^−2^ s^−1^) for 30 s in the presence of 1 µM OG, no increase in τ_2_ was observed, unlike the case after a single saturating flash. The disappearance of the OG effect was not due to the dissociation of PTOX from the thylakoid membrane during 30 s of illumination. Western blot analysis confirmed the presence of PTOX on the thylakoid membranes washed from dissociated membrane components after both single flash and continuous pre-illumination with high light (Supplementary Figure S3). The lack of the OG effect on A_fl_ kinetics in darkness after continuous illumination suggests the involvement of another process under these conditions.

### 3.4. Effect of Hydrogen Peroxide on PQ Pool Oxidation in Darkness After Illumination

We assumed that the factor affecting the A_fl_ kinetics in darkness after illumination with continuous high light is H_2_O_2_ accumulated during illumination, resulting from oxygen reduction by the PETC components. To test this hypothesis, two experimental approaches were used: adding H_2_O_2_ to thylakoid suspensions and adding catalase, an enzyme that decomposes H_2_O_2_ into water and oxygen.

The addition of catalase at a saturating concentration (250 units/mL) to the dark-adapted thylakoid suspension did not alter the shape of OJIP curves and A_fl_ value (Supplementary Figure S4). Catalase had no impact on τ_2_ when the thylakoids were pre-illuminated with a single saturating flash (Table 3), i.e., under conditions that do not lead to significant H_2_O_2_ accumulation. However, after 30 s of high light pre-illumination (650 µmol quanta m^−2^ s^−1^), when approximately 5 nmol of H_2_O_2_ accumulates in 1 mL of suspension, based on an oxygen reduction rate of 10 µmol O_2_ (mg Chl·h)^−1^ observed in [15], catalase addition led to a twofold increase in τ_2_ relative to the control, while τ_1_ remained unchanged (Table 3). Furthermore, the addition of catalase in the presence of 1 µM OG also caused a twofold increase in τ_2_ without affecting τ_1_ (Table 3). Replacing catalase with BSA, added in an equivalent amount, did not affect τ_2_, ruling out a nonspecific effect of catalase on A_fl_ kinetics.

It has been repeatedly shown that the addition of Fd to thylakoid suspensions stimulates oxygen reduction [45,46,47,48], thereby increasing the amount of H_2_O_2_ formed in the light over time. Catalase addition in the presence of Fd did not affect τ_1_ but doubled τ_2_ compared to the control (Table 3), indicating that H_2_O_2_ was formed under these conditions and was involved in the changes in A_fl_ kinetics in darkness after illumination.

Another approach involved the exogenous addition of H_2_O_2_ to the pea thylakoid suspension. In the first step, we selected a “safe” range of H_2_O_2_ concentrations that do not decrease PSII activity. We showed that H_2_O_2_ concentrations up to 0.5 mM had no significant effect on PS II activity after 60 s of incubation with H_2_O_2_ in darkness following flash illumination, while 5 mM H_2_O_2_ inhibited PS II activity by a factor of 2 (Supplementary Figure S5). Furthermore, H_2_O_2_ in concentrations above 1 mM led to the appearance of a “dip” in the I-P region in both dark-adapted and pre-illuminated thylakoids, which is not typical for thylakoids lacking an effective electron acceptor, and this “dip” greatly affected A_fl_ (Supplementary Figure S6). Thus, concentrations of H_2_O_2_ above 1 mM are unsuitable for studying PQ pool oxidation in darkness after illumination using A_fl_ kinetics measurements.

**Figure 4:**
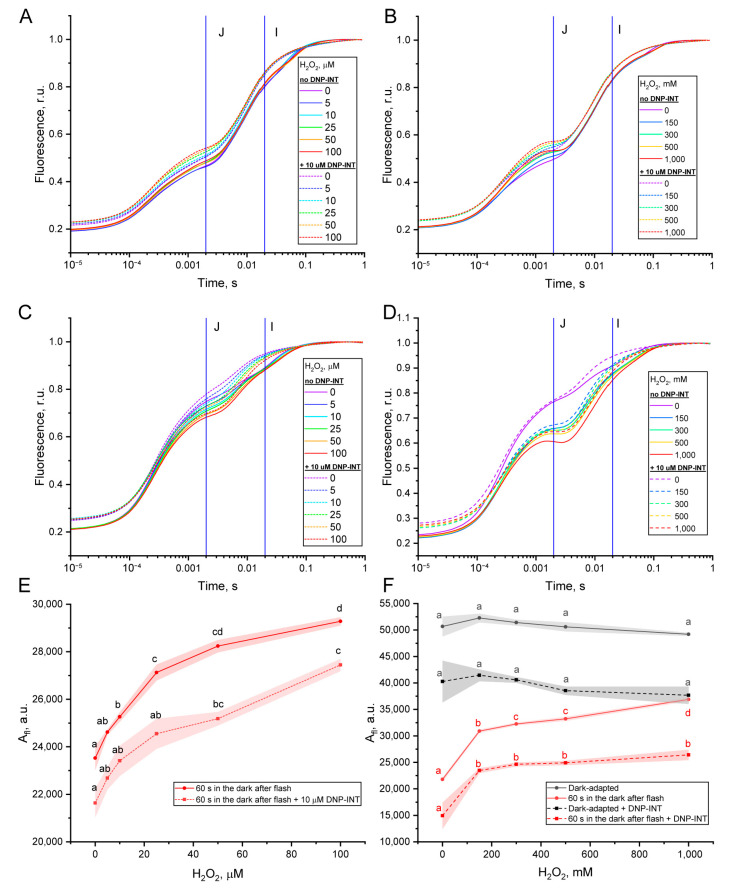
Concentration effect of H_2_O_2_ on OJIP curve shape in dark-adapted pea thylakoids (**A**,**B**) and after 60 s of darkness following illumination with a single saturating flash (**C**,**D**). Dependence of A_fl_ on H_2_O_2_ concentration in dark-adapted pea thylakoids (black line) and in pea thylakoids after 60 s of darkness following illumination with a single saturating flash (red line) (**E**,**F**). For all figures, solid lines represent conditions without DNP-INT and dashed lines represent conditions with 10 µM DNP-INT. Letters on (**E**,**F**) indicate significant difference between H_2_O_2_ concentration variants for each curve by Holm–Bonferroni test (*p* < 0.05).

The addition of H_2_O_2_ in the concentration ranges of 5 to 100 µM and 150 to 1000 µM in two independent experiments did not lead to significant changes in the shape of the OJIP curve in dark-adapted thylakoids (Figure 4A,B, solid lines), except for a slight increase in fluorescence at the J point (Fj), indicating a potential decrease in PS II activity. Despite this, no decrease in PSII activity was observed at this range of H_2_O_2_ concentrations (Supplementary Figure S5). Moreover, according to the ANOVA analysis, A_fl_ measured in dark-adapted thylakoids did not show significant changes with increasing H_2_O_2_ concentrations up to 1000 µM (Figure 4F, solid gray line).

In contrast, the OJIP curves measured after 60 s of darkness following illumination showed a strong increase in A_fl_ over the entire concentration range of H_2_O_2_ from 5 to 1000 µM (Figure 4E,F, solid red line). To determine whether the effect of H_2_O_2_ on A_fl_ was due to H_2_O_2_’s direct influence on the PQ pool or on a section of the chain downstream of the PQ pool (cytochrome b_6_f complex, plastocyanin, and PS I), we conducted experiments with H_2_O_2_ in the presence of an inhibitor of plastoquinol oxidation at the quinol-oxidizing site of the cytochrome b_6_f complex, 2,4-dinitrophenyl ether of 2-iodo-4-nitrothymol (DNP-INT) [38,49,50]. In the presence of DNP-INT, changes in A_fl_ likely indicate processes within the PQ pool and cannot be attributed to downstream acceptors. DNP-INT acts as a physical quencher of fluorescence, resulting in lower A_fl_ values compared to conditions without DNP-INT (Figure 4E,F). In the presence of DNP-INT in a saturating concentration (10 µM), according to ANOVA analysis, no changes in A_fl_ were observed in dark-adapted thylakoids as the concentration of H_2_O_2_ increased up to 1000 µM (Figure 4F, dashed gray line). However, after 60 s in the dark following flash illumination, an increase in A_fl_ was observed with increasing concentrations of H_2_O_2_ from 5 to 100 µM in the presence of DNP-INT (Figure 4E, dashed red line). These changes in A_fl_ resulted specifically from the effect of H_2_O_2_ on the J-I region, corresponding to alterations in the redox state of the PQ pool (Figure 4C,D). A further increase in H_2_O_2_ concentration did not cause statistically significant changes in A_fl_ in the presence of DNP-INT, whereas in the absence of DNP-INT, A_fl_ continued to increase (Figure 4F, solid and dashed red lines). This result indicates that 150 µM is the saturating concentration of H_2_O_2_ for PQ pool oxidation, and a further increase in H_2_O_2_ concentration raised A_fl_ in the absence of DNP-INT due to the influence of H_2_O_2_ on the PETC components downstream of the PQ pool. Thus, experiments in the presence of DNP-INT demonstrated that low concentrations of H_2_O_2_ (5–100 µM) are sufficient to accelerate PQ pool oxidation in darkness after illumination.

## 4. Discussion

In this study, the pathways of oxidation of the PQ pool in darkness after illumination were thoroughly investigated. The dependence of the area above the OJIP fluorescence curve (A_fl_) on time intervals after illumination was used to analyze PQ pool oxidation. This approach is widely used [2,36,37]. However, this is an indirect method of measuring the PQ pool redox state, as the magnitude of A_fl_ reflects the redox state of Q_A_, which can be influenced by electron transfer across the entire PETC. Therefore, it is inaccurate to directly attribute changes in A_fl_ kinetics exclusively to processes occurring within the PQ pool without additional measurements. For instance, we observed that in thylakoids after 60 s in darkness after illumination with a saturating pulse, the addition of H_2_O_2_ at high concentrations caused a “dip” in fluorescence in the I-P region of the OJIP curve, which is related to processes in the PETC components downstream of the PQ pool (Supplementary Figure S6). This “dip” inevitably resulted in an increase in A_fl_, which can be erroneously interpreted as higher PQ pool oxidation by H_2_O_2_. To avoid misinterpretation, we applied DNP-INT, which blocks electron transfer from the PQ pool to downstream acceptors. The changes in A_fl_ in the DNP-INT-treated thylakoids after 60 s in darkness after a saturating flash can be interpreted more reliably as evidence of a direct reaction of the PQ pool with H_2_O_2_. Thus, this key finding indicates that under our experimental conditions, changes in A_fl_ kinetics predominantly reflect processes within the PQ pool.

The biphasic curves of A_fl_ kinetics were analyzed by applying a fitting model to calculate the τ values for the “fast” and “slow” phases (τ_1_ and τ_2_). The biphasic nature of PQ pool oxidation in darkness has been previously demonstrated using both fluorescence and optical methods in isolated thylakoids and intact leaves [2,35,36]. The “fast” phase of oxidation is consistent with the oxidation of the PQ pool by downstream acceptors of PETC [51] and correlates with the presence of efficient electron acceptors from PS I, resulting in a higher level of P_700_^+^ in the light (Figure 1). As shown in experiments conducted under anaerobic and aerobic conditions with isolated thylakoids and chloroplasts (Figure 2), the “slow” phase corresponds to the oxygen-dependent pathway of PQ pool oxidation. The specific roles of PTOX and H_2_O_2_ in facilitating oxygen-dependent PQ pool oxidation in darkness were examined, aiming to clarify their relevant contributions to this process.

To identify the role of PTOX, a specific inhibitor, octyl gallate (OG), was used. Gallic acid derivatives have shown high specificity and effectiveness toward recombinant PTOX [44]. Octyl gallate has an IC_50_ of 0.4 µM, and 5 µM OG fully inhibits PTOX activity, while at 1 µM OG, PTOX activity decreases by 80% [44]. We selected 1 µM OG to assess the contribution of PTOX to PQ pool oxidation in thylakoid membranes while minimizing side-effects on the OJIP curve shape, which occurs at concentrations of 5 µM OG and higher (Figure 3A,B). In the presence of 1 µM OG, the “slow” phase of PQ pool oxidation slows down twofold compared to the control after a single flash illumination (Table 2). This suggests that in plants, PTOX can effectively oxidize the PQ pool in darkness. However, continuous pre-illumination for 30 s with high light completely eliminated the effect of OG on PQ pool oxidation kinetics (Table 2). This result could have been explained by PTOX dissociation from the thylakoid membrane caused by the illumination reported earlier [52]. However, western-blot analysis showed that the binding of PTOX to the thylakoid membrane persisted after illumination under our conditions (Supplementary Figure S3). One may also assume that PTOX activity may change due to the reduction in its regulatory cysteine residues, as it is known that PTOX activity is regulated by stromal thioredoxins, which deactivate PTOX in the light, while in darkness, the oxidation of thiols leads to PTOX reactivation within 15 min [53]. However, we used isolated thylakoids lacking all water-soluble stromal components, including thioredoxins, so the light-dependent modulation of PTOX activity through regulatory cysteines was excluded. Therefore, the absence of the effect of OG on τ_2_ in the case of continuous high light pre-illumination strongly suggests the involvement of another factor affecting PQ pool oxidation.

Indeed, experiments with varying pre-illumination conditions indicate that after continuous high light pre-illumination, the “slow” phase of A_fl_ kinetics accelerates compared to single saturating flash pre-illumination (Table 1). Additionally, increasing the intensity of continuous pre-illumination led to a substantial acceleration of the “slow” phase of A_fl_ kinetics. In our experimental system, O_2_ was the only electron acceptor from the PETC, and its reduction resulted in H_2_O_2_ accumulation. We suggested that H_2_O_2_ accumulation during continuous pre-illumination accelerated PQ pool oxidation in darkness, as previously assumed [3]. The involvement of other ROS generated by the PETC appears unlikely, although PQH_2_ molecules were shown to be oxidized by superoxide anion radicals [3] and singlet oxygen [4,33]. The short lifetime of these ROS precludes their accumulation and minimizes their contribution to the “slow” phase of A_fl_ kinetics, which begins 1–3 s into darkness, although superoxide anion radicals could partially contribute to the “fast” phase, as proposed in [35]. In contrast, H_2_O_2_ has a relatively long lifetime and can accumulate in thylakoid suspension.

Catalase, which prevents the accumulation of H_2_O_2_ in the thylakoid suspension under illumination, increased τ_2_ twofold after continuous illumination but had no effect on τ_2_ after single flash illumination (Table 3). Moreover, the effect of catalase on the “slow” phase of A_fl_ kinetics was observed in the presence of Fd (Table 3), which induces higher H_2_O_2_ accumulation through its activity and increases oxygen reduction by membrane-bound PETC components [47,48], including phyllosemiquinone [54,55]. One might speculate that H_2_O_2_ regulates PTOX activity through oxidation of regulatory cysteines; however, the effect of catalase was observed even when PTOX was inhibited with OG (Table 3), indicating that H_2_O_2_ acts directly on the PQ pool, rather than on PTOX.

In experiments involving H_2_O_2_ addition, a clear concentration-dependent effect on the OJIP curves and the derived A_fl_ values was observed (Figure 4A–F). To isolate the effect of H_2_O_2_ on the PQ pool and eliminate its influence on downstream acceptors, we used DNP-INT, an inhibitor of PQH_2_ oxidation in the cytochrome b_6_f complex. Indeed, in the DNP-INT-treated thylakoids, A_fl_ in darkness after illumination increased with increasing of H_2_O_2_ concentration to 100–150 µM, indicating that H_2_O_2_ primarily affects the PQ pool oxidation (Figure 4E,F, dashed red line). This range corresponds to physiological concentrations of H_2_O_2_ detected in chloroplasts under stress conditions. A further increase in H_2_O_2_ concentration did not result in A_fl_ changes in the DNP-INT-treated thylakoids, in contrast to thylakoids in the absence of DNP-INT (Figure 4F, solid and dashed red lines), in which A_fl_ changes were likely associated with the effect of H_2_O_2_ not only on the PQ pool but also on downstream PETC components.

Thus, our results (Table 3 and Figure 4A–F) indicate that H_2_O_2_ interacts with PQ pool components, accelerating its oxidation. For a long time, H_2_O_2_ formed in chloroplasts was considered only as a destructive and inhibitory agent. Later, the role of H_2_O_2_ as a signaling agent in retrograde signaling became evident [7]. Growing evidence suggests that H_2_O_2_ in chloroplasts plays a regulatory role, namely, it activates NDH-dependent cyclic electron flow around PS I [56]; downregulates STT7 and STN7 kinases [57,58], key enzymes performing state transitions in green algae and higher plants; downregulates zeaxanthin epoxidase activity [59]; and activates LHCSR3-dependent nonphotochemical quenching in *Chlamydomonas reinhardtii* [60]. H_2_O_2_ has a relatively long lifetime and can even diffuse over long distances, such as out of chloroplasts [39,61]. Our data indicate that H_2_O_2_ can be considered as a metabolic oxidizer of the PQ pool in plants.

However, the mechanism by which H_2_O_2_ oxidizes the PQ pool remains unknown. Previously, Khorobrykh and Tyystjärvi demonstrated the oxidation of PQH_2_ molecules by H_2_O_2_ in methanol [14]. In that work, incubating 75 µM PQH_2_ with 5 mM H_2_O_2_ in methanol led to the oxidation of only 25% of PQH_2_ within the first 2 min, while longer incubation did not result in further PQH_2_ oxidation. The authors of that study concluded that H_2_O_2_ likely deprotonates the PQH_2_ molecules, facilitating their subsequent reaction with O_2_. However, due to the rapid accumulation of H_3_O_2_^+^, equilibrium is quickly reached, preventing further deprotonation of PQH_2_ by H_2_O_2_. Khorobrykh and Tyystjärvi suggested that H_2_O_2_ acts more as a catalyst in the oxidation of PQH_2_ than as a full-fledged oxidizer [14]. In contrast, [34] proposed that H_2_O_2_ molecules can react directly with semiquinones, forming a hydroxyl radical and a hydroxide ion, a process the authors termed the metal-independent Fenton reaction:

Q^●−^ + H_2_O_2_ → Q + OH^●^ + OH^−^ (Reaction 1)

However, the rate constant for this reaction of semiquinones with H_2_O_2_ is approximately 10^4^ m^−1^s^−1^, significantly lower than the rate constant for semiquinones reacting with O_2_, which is around 10^8^ m^−1^s^−1^ [62]. Our results do not yet clarify the specific mechanism by which H_2_O_2_ oxidizes the PQ pool in thylakoid membranes. This question requires further investigation.

Considering the effects of octyl gallate, H_2_O_2_, and catalase under various pre-illumination conditions on PQ pool oxidation in darkness, it appears that in vivo the PQ pool oxidation pathways in darkness, mediated by PTOX and H_2_O_2_, operate depending on the light exposure history. A single saturating flash or continuous low light does not lead to a high amount of H_2_O_2_ accumulation in chloroplasts, whereas under these conditions, regulatory cysteines of PTOX remain oxidized and, thus, PTOX remains active, facilitating PQ pool oxidation. High light exposure inactivates PTOX by reducing its regulatory cysteines through thioredoxins and simultaneously leads to H_2_O_2_ accumulation, which can efficiently oxidize the PQ pool in darkness and potentially accelerate PTOX reactivation by oxidizing its regulatory cysteines. Therefore, these pathways of PQ pool oxidation in darkness allow plants to adjust the redox state of the PQ pool and prevent over-reduction under changing light conditions, which may be one of the mechanisms for protecting the photosynthetic apparatus from photoinhibition.

## 5. Conclusions

This study demonstrates that both PTOX and the H_2_O_2_, generated by the PETC in thylakoids under light, contribute to PQ pool oxidation in darkness. Since PTOX activity decreases under high light intensity due to the reduction in its regulatory cysteine residues by thioredoxins, PTOX predominantly contributes to PQ pool oxidation in darkness following exposure to low light intensities. Under stress conditions, such as high light exposure, when PTOX activity is downregulated, the production of hydrogen peroxide in chloroplasts, including its “membrane-associated” fraction, increases. As a result, hydrogen peroxide assumes a more prominent role in PQ pool oxidation in darkness under these conditions.

## Figures and Tables

**Table 1:** Effect of pre-illumination type and light intensity on τ_1_ and τ_2_ values, calculated from A_fl_ dependencies on time intervals in darkness after illumination of pea thylakoids, ±SE. Medium: 0.1 M sucrose, 1 µM GrD. Data rows marked with different letters indicate statistically significant differences at *p* < 0.05, according to a *t*-test. Bold lines separate different experiments.

Pre-Illumination Conditions	τ_1_, s	τ_2_, s
Single saturating flash pre-illumination (1.5 s with 3000 µmol quanta m^−2^ s^−1^)	0.95 ± 0.08 ^a^	80 ± 10 ^a^
Continuous pre-illumination for 30 s with 650 µmol quanta m^−2^ s^−1^	0.90 ± 0.07 ^a^	53 ± 6 ^b^
Continuous pre-illumination for 30 s with 10 µmol quanta m^−2^ s^−1^	2.56 ± 0.48 ^a^	108 ± 7 ^a^
Continuous pre-illumination for 30 s with 1000 µmol quanta m^−2^ s^−1^	2.57 ± 0.64 ^a^	79 ± 8 ^b^

**Table 2:** Effect of octyl gallate (OG) on τ_1_ and τ_2_ values, calculated from A_fl_ dependencies on time intervals in darkness after illumination of pea thylakoids with a single saturating flash or continuous light, ±SE. Medium: 0.1 M sucrose, 1 µM GrD. Data rows marked with different letters indicate statistically significant differences at *p* < 0.05, according to a *t*-test. Bold lines separate different experiments.

Pre-Illumination Conditions	Addition	τ_1_, s	τ_2_, s
Single saturating flash pre-illumination (1.5 s with 3000 µmol quanta m^−2^ s^−1^)	-	1.01 ± 0.19 ^a^	64 ± 5 ^a^
	1 uM OG	1.41 ± 0.23 ^a^	181 ± 16 ^b^
Continuous pre-illumination for 30 s with 650 µmol quanta m^−2^ s^−1^	-	1.66 ± 0.38 ^a^	100 ± 11 ^a^
	1 uM OG	0.98 ± 0.26 ^a^	92 ± 12 ^a^

**Table 3:** Effect of catalase on τ_1_ and τ_2_ values, calculated from A_fl_ dependencies on time intervals in darkness after illumination of pea thylakoids with a single saturating flash or continuous high light, ±SE. Medium: 0.1 M sucrose, 1 µM GrD. Data rows marked with different letters indicate statistically significant differences at *p* < 0.05, according to a *t*-test. Bold lines separate different experiments.

Pre-Illumination Conditions	Addition	τ_1_, s	τ_2_, s
Continuous pre-illumination for 30 s with 650 µmol quanta m^−2^ s^−1^	-	1.5 ± 0.2 ^a^	66 ± 8 ^a^
	250 U/mL catalase	1.5 ± 0.2 ^a^	136 ± 27 ^b^
	1 uM OG	0.66 ± 0.07 ^a^	62 ± 4 ^a^
	1 uM OG + 250 U/mL catalase	0.86 ± 0.19 ^a^	122 ± 22 ^b^
	15 uM Fd	0.31 ± 0.12 ^a^	56 ± 5 ^a^
	15 uM Fd + 250 U/mL catalase	0.36 ± 0.09 ^a^	102 ± 8 ^b^
Single saturating flash pre-illumination (3000 µmol quanta m^−2^ s^−1^)	-	0.75 ± 0.06 ^a^	111 ± 5 ^a^
	250 U/mL catalase	0.65 ± 0.05 ^a^	90 ± 4 ^b^

## Data Availability

The raw data supporting the conclusions of this article will be made available by the authors on request.

## Supplementary Materials

The following supporting information can be downloaded at: https://www.mdpi.com/article/10.3390/plants13243479/s1, Figure S1. OJIP curves measured in isolated pea thylakoids in the presence of 15 µM Fd and 500 µM NADP^+^ at the indicated time intervals in darkness after illumination with a single saturating flash (**A**) and continuous light at 650 µmol quanta m^−2^s^−1^ for 30 s (**B**) (specific time points are indicated); Figure S2. Dependence of the electron transport rate, measured in a pea thylakoid suspension in the presence of 1 µM GrD and 50 µM methyl viologen, on OG concentration; Figure S3. Immunoblots of PTOX protein after denaturing electrophoresis of Arabidopsis thylakoids (from left to right): untreated, treated in the dark for 60 s, treated under saturating flash (3000 μmol quanta m^−2^s^−1^) for 1.5 s, treated under high light (approx. 650 μmol quanta m^−2^s^−1^) for 30 s; Figure S4. OJIP curves measured in the dark-adapted pea thylakoids in the presence (dashed line) and in the absence (solid line) of 250 U/mL catalase; Figure S5. PS II activity in pea thylakoids, measured in the presence of potassium ferricyanide and DCBQ as electron acceptors, after incubating thylakoids with different concentrations of H_2_O_2_ for 60 s in darkness after illumination with a single saturating flash; Figure S6. Concentration effect of H_2_O_2_ on OJIP curve shape in dark-adapted pea thylakoids (**A**) and in pea thylakoids after 60 s of darkness following illumination with a single saturating flash (**B**). (**C**) Dependence of A_fl_ on H_2_O_2_ concentration in dark-adapted pea thylakoids (black line) and in pea thylakoids after 60 s of darkness following illumination with a single saturating flash (red line).

## Abbreviations

A_fl_	area above OJIP curve
DNP-INT	2,4-dinitrophenyl ether of 2-iodo-4-nitrothymol
PQ: PQH_2_: PQ^●−^	plastoquinone, plastohydroquinone, plastosemiquinone
PETC	photosynthetic electron transport chain
ROS	reactive oxygen species
PS I, PS II	photosystem I, photosystem II respectively
Chl	chlorophyll *a*
Fd	ferredoxin
PTOX	plastid terminal oxidase
OG	octyl gallate
