# Supplementary material for: Pathways of Oxygen-Dependent Oxidation of the Plastoquinone Pool in the Dark After Illumination
Source: Plants (Basel). 2024 Dec 12;13(24):3479. doi: 10.3390/plants13243479 (PMC11678207; doi:10.3390/plants13243479)
Supplement: Supplementary file 1 [file plants-13-03479-s001.zip › plants-3322547-supplementary.pdf]

Supplementary

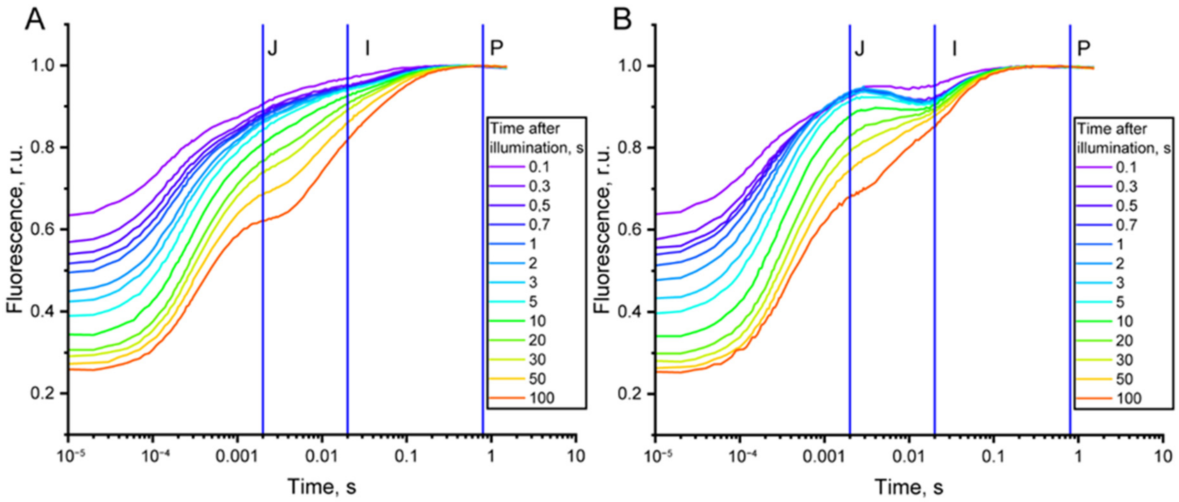

Suppl. Figure S1 – OJIP curves measured in isolated pea thylakoids in the presence of 15 μM Fd and 500 μM $NADP^+$ at the indicated time intervals in darkness after illumination with a single saturating flash (A) and continuous light at 650 μmol quanta $m^{-2}s^{-1}$ for 30 s (B) (specific time points are indicated)

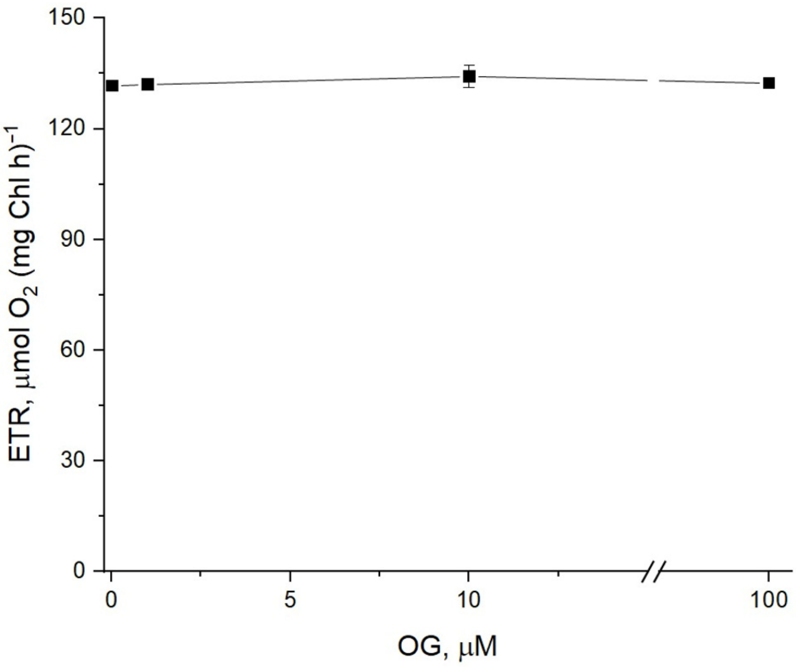

Suppl. Figure S2 – Dependence of the electron transport rate, measured in a pea thylakoid suspension in the presence of 1 μM GrD and 50 μM methyl viologen, on OG concentration.

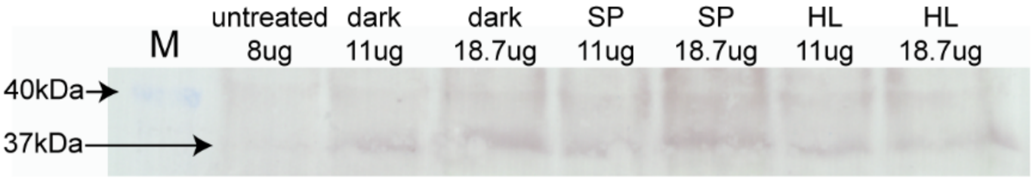

Suppl. Figure S3 – Immunoblots of PTOX protein after denaturing electrophoresis of Arabidopsis thylakoids (from left to right): untreated, treated in the dark for 60 s, treated under saturating flash (3000 μmol quanta $m^{-2}s^{-1}$) for 1.5 s, treated under high light (approx. 650 μmol quanta $m^{-2}s^{-1}$) for 30 s. Samples containing denoted content (μg) of chlorophyll were loaded onto a 15% polyacrylamide gel (PAAG) for separation.

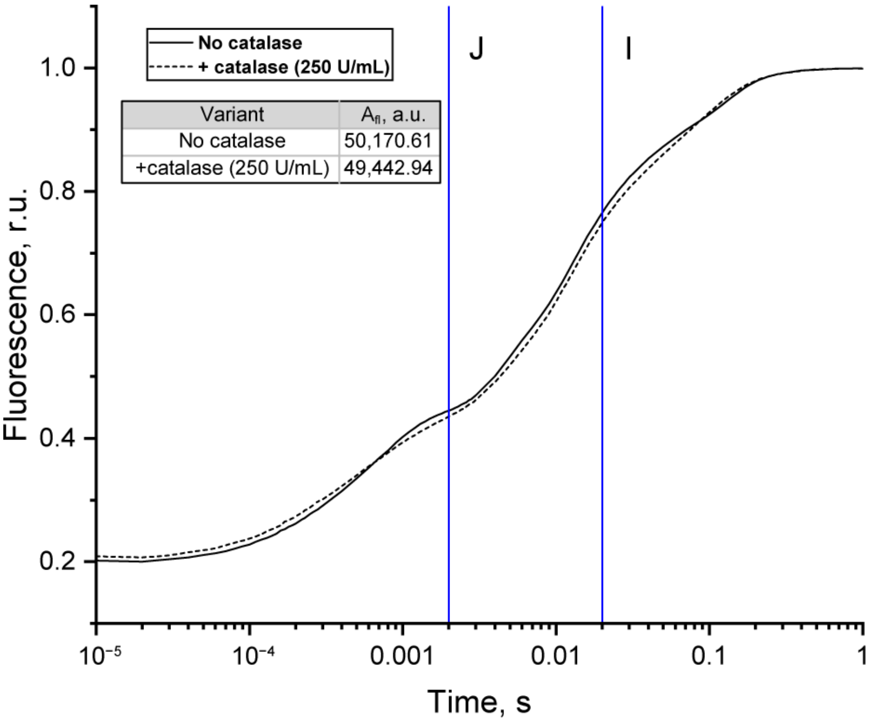

Suppl. Figure S4 – OJIP curves measured in the dark-adapted pea thylakoids in the presence (dashed line) and in the absence (solid line) of 250 U/mL catalase. A_{fl} values for both variants are presented in the table.

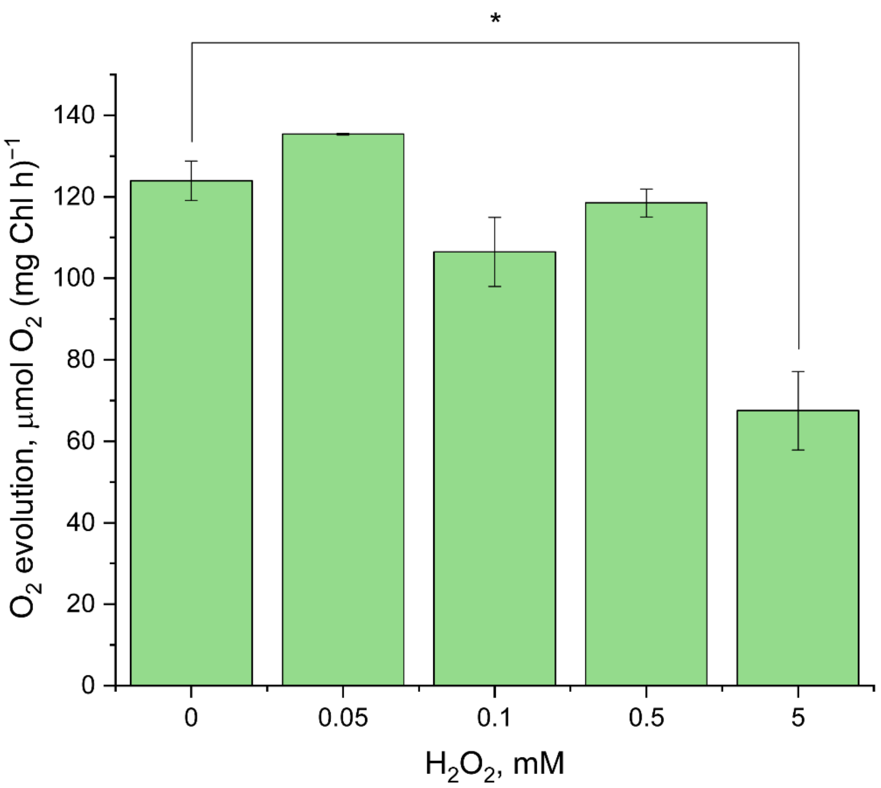

Suppl. Figure S5 – PS II activity in pea thylakoids, measured in the presence of potassium ferricyanide and DCBQ as electron acceptors, after incubating thylakoids with different concentrations of H_2O_2 for 60 s in darkness after illumination with a single saturating flash. Asterisks denotes significantly different values, according to ANOVA with Holm-Bonferroni ($p<0.05$)

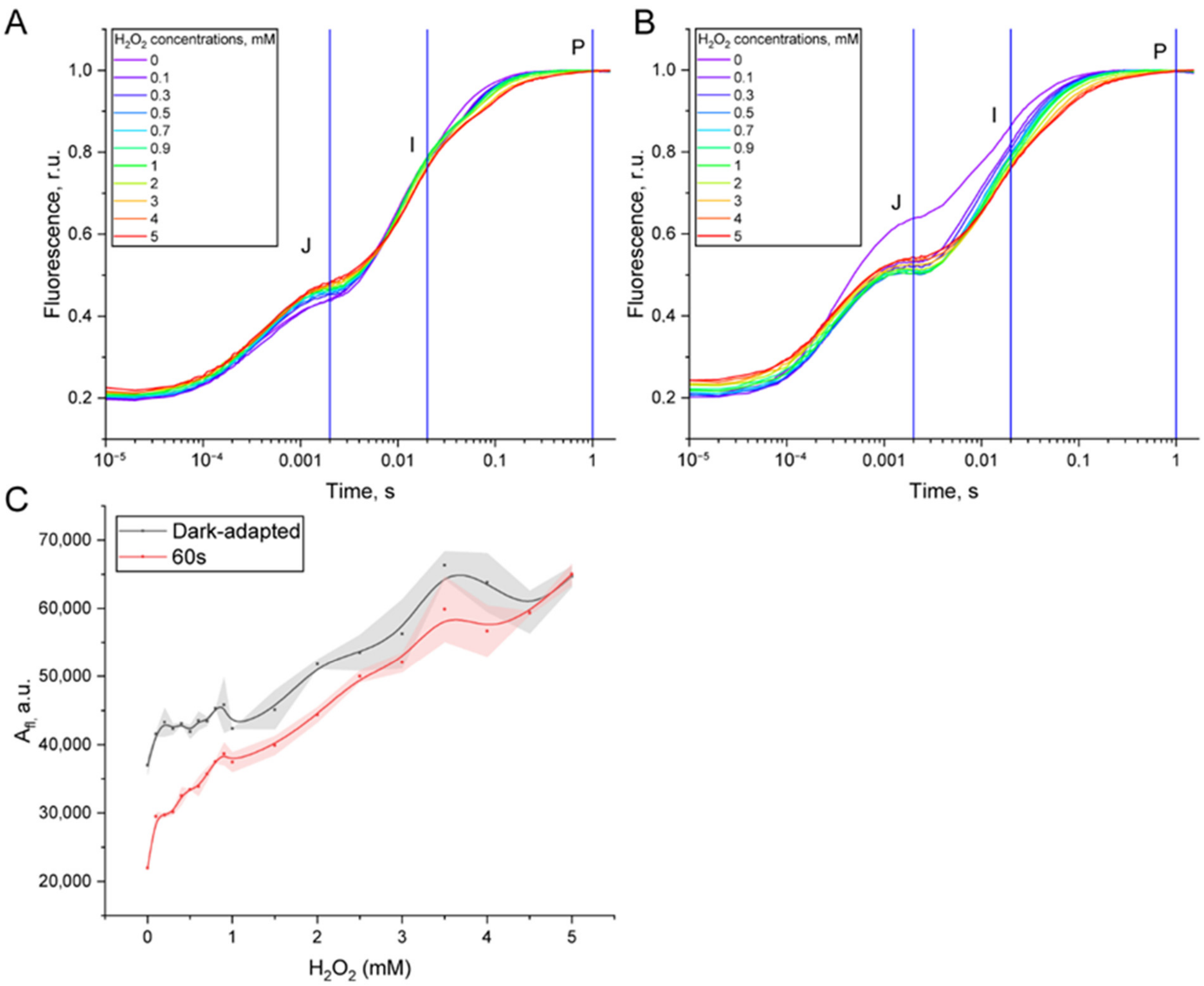

Suppl. Figure S6 – Concentration effect of H_2O_2 on OJIP curve shape in dark-adapted pea thylakoids (A) and in pea thylakoids after 60 s of darkness following illumination with a single saturating flash (B). (C) – Dependence of A_{fl} on H_2O_2 concentration in dark-adapted pea thylakoids (black line) and in pea thylakoids after 60 s of darkness following illumination with a single saturating flash (red line).
